# Nitric Oxide Synthase 2 Improves Proliferation and Glycolysis of Peripheral γδ T Cells

**DOI:** 10.1371/journal.pone.0165639

**Published:** 2016-11-03

**Authors:** Laetitia Douguet, Julien Cherfils-Vicini, Lloyd Bod, Renée Lengagne, Eric Gilson, Armelle Prévost-Blondel

**Affiliations:** 1 INSERM, U1016, Institut Cochin, Paris, France; 2 CNRS, UMR8104, Paris, France; 3 Université Paris Descartes, Sorbonne Paris Cité, Paris, France; 4 Institut de Recherche sur le cancer et le vieillissement, CNRS UMR7284, INSERM U1081, Université de Nice, Nice, France; 5 Département de génétique médicale, Hôpital l’Archet, CHU de Nice, Nice, France; Center for Cancer Research, UNITED STATES

## Abstract

γδ T cells play critical roles in host defense against infections and cancer. Although advances have been made in identifying γδ TCR ligands, it remains essential to understand molecular mechanisms responsible for *in vivo* expansion of γδ T cells in periphery. Recent findings identified the expression of the inducible NO synthase (NOS2) in lymphoid cells and highlighted novel immunoregulatory functions of NOS2 in αβ T cell differentiation and B cell survival. In this context, we wondered whether NOS2 exerts an impact on γδ T cell properties. Here, we show that γδ T cells express NOS2 not only *in vitro* after TCR triggering, but also directly *ex vivo*. Nos2 deficient mice have fewer γδ T cells in peripheral lymph nodes (pLNs) than their wild-type counterparts, and these cells exhibit a reduced ability to produce IL-2. Using chemical NOS inhibitors and Nos2 deficient γδ T cells, we further evidence that the inactivation of endogenous NOS2 significantly reduced γδ T cell proliferation and glycolysis metabolism that can be restored in presence of exogenous IL-2. Collectively, we demonstrate the crucial role of endogenous NOS2 in promoting optimal IL-2 production, proliferation and glycolysis of γδ T cells that may contribute to their regulation at steady state.

## Introduction

Nitric oxide (NO) is a short half-life molecule that can diffuse through the membranes and act in an autocrine or paracrine manner [1]. The production of NO is catalyzed by three distinct isoforms of nitric oxide synthase (NOS) from the amino-acid arginine. Nitric oxide synthase 2 (NOS2) is the inducible form of these enzymes and its expression is found in various cell types such as myeloid, stromal and tumor cells [2–7]. Recent studies established that primary B and αβ T lymphocytes could also express NOS2 after IL-6 stimulation or T cell receptor (TCR) triggering respectively [8–11]. While NOS2 is required for the survival of plasma cells [10], two opposite effects were described on activated CD4^+^ αβ T cells. Autocrine NOS2 inhibits the differentiation of murine CD4^+^ T cells into IL-17-producing T helper cells (Th17) [8], whereas it is required for inducing and maintaining Th17 phenotype in cells derived from human CD4^+^ T lymphocytes [9].

Unlike αβ T cells, γδ T cells develop early in ontogeny [12,13] and most of them do not recognized peptides bound to conventional MHC molecules [14–17]. While advances have been made in characterizing γδ TCR ligands, little is known about their activation processes in periphery, thus limiting the use of their functional properties in therapy. Due to their pleiotropic functions, γδ T cells exhibit key roles in host defense against infection, sterile stress and cancer [18,19]. γδ T cells could directly lyse target cells through the production of granzymes, cooperate with αβ T, B and dendritic cells and also secrete a large range of chemokines and cytokines (i.e. IFN-γ or IL-17). Indeed, their IFN-γ secretion triggers protective responses to the West Nile virus infection [20] and tumor immunity [21–23], whereas their IL-17 production is important to control various bacterial infections [24,25].

While NOS2 expression affects αβ T cell differentiation and plasmocyte survival, its potential impact on γδ T cells at steady state remains unexplored. The aim of this study is to explore the role of NOS2 on γδ T cell functions. To raise this issue, we analyzed the pool of γδ T cells in wild-type (WT) and Nos2-deficient (Nos2KO) mice. In order to unravel how NOS2 regulates the peripheral γδ T cell pool *in vivo*, we used γδ T cells, either competent or deficient for NOS2, in presence or not of chemical NOS inhibitors. We compared their capacity to proliferate *in vitro* and investigated for the first time their metabolism.

## Materials and Methods

### Mice and Ethics statement

C57BL/6J (designated as WT) mice were purchased from Harlan and Jackson Laboratories. C57BL/6J *Nos2*^*-/-*^ (designated as Nos2KO) described elsewhere [26] were kindly provided by Dr. H-J Garchon (Inserm U1173 and University of Versailles Saint-Quentin, France) and bred locally. WT and Nos2KO mice were euthanized at 6 to 12 weeks of age by cervical elongation. The animal experiment protocol approval number is CEEA34.AB.038.12 and was delivered by the Institutional Animal Ethics Committee of the Descartes University of Paris. All mice were maintained under specific pathogen-free conditions in our animal facility which also received an approval number (A75-14-02).

### Single cell suspension procedures

LNs and thymus were mechanically dissociated, homogenized, and passed through a 100 μM cell strainer in 5% (vol/vol) FCS and 0.5% EDTA in phosphate-buffered saline (PBS). For skin suspensions, ears were collected and cut in small parts and digested with 0.4 mg ml^-1^ liberase, 0.05 mg ml^-1^ collagenase D, and 0.1 mg ml^-1^ DNase I (Roche) for 1h at 37°C.

### Culture of γδ T cells

γδ T cells were sorted from pLNs. CD4^+^, CD8^+^ and CD19^+^ cells were depleted using Dynabeads (Invitrogen) before a negative sorting using Aria III cytometer (BD Biosciences). Highly purity of γδ T cells with untouched TCR was obtained. γδ T cells were cultured in RPMI 1640+Glutamax (Gibco) with 10% FCS, 100 U ml^-1^ penicillin and streptomycin, 10 mM HEPES, 1 mM sodium pyruvate, non-essential amino acids, 50 μM 2-mercaptoethanol in 96 well plates at 37°C, 5% CO_2._ When indicated 15–30 U ml^-1^ of rIL-2 and 15 μg ml^-1^ rIL-7 (R&D Systems) were used. Cells were cultured on plate-bound with 0.1 μg ml^-1^ anti-CD3ε (145-2C11) and 10 μg ml^-1^ anti-CD28 (37.51) (both from eBioscience).

### Antibodies

Following anti-mouse Abs were used for cytometry analysis and cell sorting: FITC–conjugated anti-B220 (RA3-632), PE–conjugated anti-δ TCR (GL3) and anti-NK1.1 (PK136), APC- conjugated anti-CD45.2 (104), anti-IL-2 (JES6-5H4), PerCP-Cy5.5 –conjugated anti-CD3 (145-2C11), anti-β TCR (H57-597), and anti-CD45.2 (104), Pacific Blue- conjugated anti-CD4 (RM4-5), APC-H7-conjugated anti-CD8 (53–6.7). Abs were purchased from BD Biosciences except anti-B220 and anti-β TCR from eBioscience. NOS2 staining was performed using a primary goat anti-mouse Ab (M19 Santa Cruz) following by anti-goat PerCP conjugated Ab (Jackson immuno research).

Following purified anti-mouse Abs were purchased from eBioscience and used to deplete cells before γδ T cells cell sorting: anti-CD19 (eBio1D3), anti-CD8 (53–6.7), anti-CD4 (GK 1.5).

Microscopy was performed using primary Abs; purified hamster anti-mouse γδ TCR (GL3, BD Biosciences) and rabbit anti-mouse NOS2 (Calbiochem). Alexa fluor 488-conjugated goat anti-rabbit (Jackson immuno research), and Alexa fluor 647-conjugated goat anti-hamster (Biolegend) were used as secondary Abs.

### Cell staining and flow cytometry

Surface staining was performed by incubating cells on ice, for 20 min, with saturating concentrations of labeled Abs in PBS, 5% FCS and 0.5% EDTA. Mouse cell-staining reactions were preceded by a 15-min incubation with purified anti-CD16/32 Abs (FcγRII/III block; 2.4G2) obtained from hybridoma supernatants. Intracellular cytokine staining were performed after stimulation of single cell suspensions with Phorbol 12-myristate 13-acetate (PMA) (50 ng ml^-1^) (Sigma), ionomycin (0.5 μg ml^-1^) (Sigma) and 1 μL ml^-1^ Golgi Plug (BD Biosciences) for 4h at 37°C 5% CO_2_. Cells were incubated with Live/Dead Blue stain (Invitrogen), according to the manufacturer protocol prior to Ab surface staining. Then, intracellular staining was performed using Cytofix/Cytoperm kit (BD biosciences) following the manufacturer’s instructions. γδ T cell apoptosis were assessed *ex vivo* by staining pLNs for FITC-conjugated annexin V (BioLegend) according to the manufacturer’s instructions. Data files were acquired and analyzed on LSRII using Diva software (BD Biosciences).

### Microscopy

First, single cell suspensions from pLNs were incubated for 15 min with purified anti-CD16/32 Abs. γδ T cell staining was performed by incubating cells with purified hamster anti-mouse γδ TCR for 20 min at 4°C. After washes, cells were incubated with the goat anti-hamster Ab for 20 min at 4°C. Then, intracellular NOS2 staining was performed using Cytofix/Cytoperm kit. Cells were incubated with anti-NOS2 Ab overnight at 4°C. After washes, cells were incubated with the goat anti-rabbit Ab for 20 min at 4°C. Then cells were centrifuged onto a microscope slide using Cellspin 1 (Tharmac). Slides were washed in PBS before being labeled with DAPI. Slides were mounted in Vectashield mounting medium (Vector Labs). Images were acquired using an automated high-resolution scanning system (Lamina, PerkinElmer) with 40X objective. Images were analyzed with Pannoramic Viewer (3DHISTECH).

### Functional assays

For γδ T cell proliferation, cells were labeled with 2.5 μM CFSE (Molecular Probes) at 37°C for 7 min. Cells were seeded in a 96-well plate pre-coated with anti-CD3ε and anti-CD28. After incubation at 37°C for 48h, cells were stained with Live/Dead Blue Stain (Invitrogen) before being analyzed by flow cytometry for CFSE dilution. When indicated 15 U ml^-1^ rIL-2 (R&D Systems) or 10 mM L-NMMA (Calbiochem), 0.5 mM N6-(1-iminoethyl)-l-lysine dihydrochloride (L-NIL, Calbiochem) were added to the cultures.

For metabolic assays, γδ T cells were sorted from WT and Nos2KO mice and cultured for 4 days in 0.1 μg ml^-1^ anti-CD3ε, 10 μg ml^-1^ anti-CD28, 15 μg ml^-1^ rIL-7 and 15 U ml^-1^ rIL-2. Metabolism analyses were performed directly or after 18h of resting followed by 4h of stimulation in media containing 5 mM L-NMMA and or 15 U ml^-1^ rIL-2 when indicated. 5.10^5^ cells per well were plated in Seahorse plates coated with CellTak (Corning). The OCR and ECAR were measured in XF medium (unbuffered RPMI containing 2 mM glutamine, pH 7.4) under basal conditions and in response to glucose (25 mM), oligomycin (1 μM), FCCP (1.5 M) plus pyruvate (1 mM) and antimycin A (1 μM) plus rotenone (0.1 μM) with an XF-24 Extracellular Flux Analyzer (Seahorse Bioscience). Rate of glycolysis was defined as the difference between ECAR following the injection of glucose and the basal ECAR reading.

### Protein quantification

Quantitative determination of IL-2 were performed in culture supernatants during γδ T cell proliferation experiments using kit from R&D Systems according to supplier instructions.

### Statistics

Data are expressed as mean ± SEM. The significance of differences between two series of results was assessed using the Mann-Whitney test. (*, p < 0.05; **, p < 0.01; ***, p < 0.001). All statistical analyses were performed using Prism 5 software (GraphPad).

## Results

### NOS2 regulates the pool of peripheral lymph node γδ T cells *in vivo*

**Fig 1:**
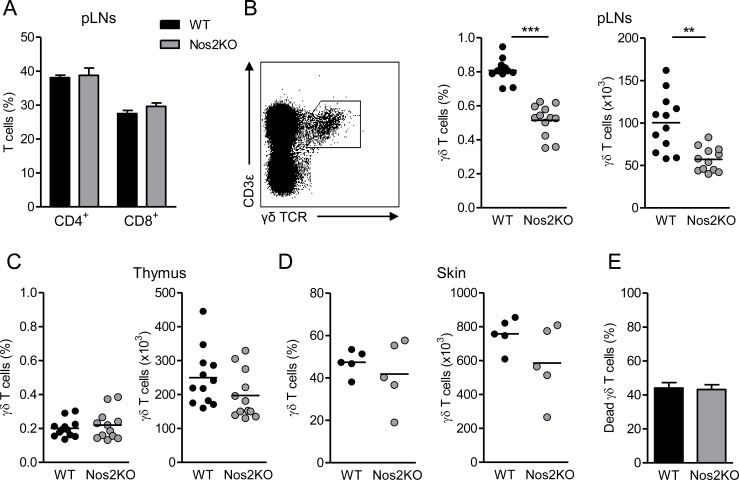
Influence of NOS2 deficiency on γδ T cells *in vivo*. (A) Percentages of CD4^+^ and CD8^+^ αβ T cells from pLNs of WT (n = 12) and Nos2KO (n = 12) mice. (B-D) Representative dot plot of peripheral γδ T cells defined as CD3ε^+^TCRγδ^+^ cells (B—left). Percentages and absolute numbers of γδ T cells from pLNs (B), thymus (C) and skin (D) of WT (n = 12, except for E n = 5) and Nos2KO (n = 12, except for E n = 5) mice. (E) Proportion of dead γδ T cells from pLNs of WT (n = 10) and Nos2KO (n = 9) mice analyzed *ex vivo* by annexin V staining. Data are pooled from one (D), two (B, C) or three (A, E) experiments. Bars are mean or mean ± SEM and each point represents one mouse. ** *p<0*.*01*, *** *p<0*.*001* (Mann-Whitney’s test).

**Fig 2:**
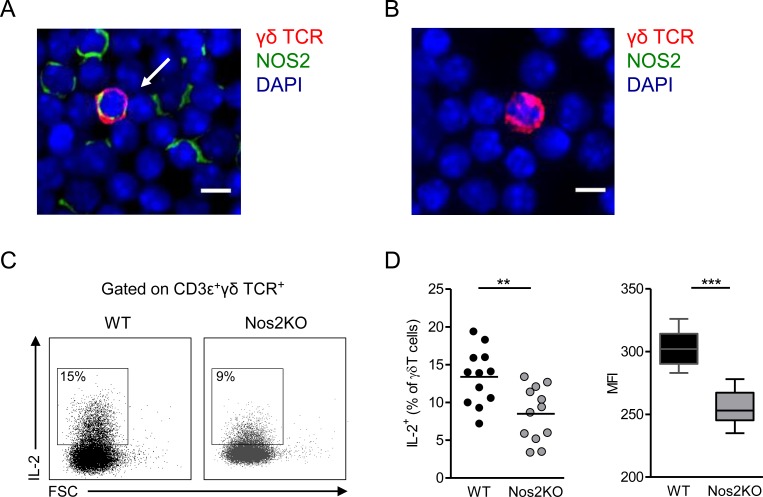
Effect of NOS2 deficiency on IL-2 production by pLNs γδ T cells. (A-B) Representative microscopy images showing a γδ T cell positive (A) or negative (B) for NOS2 derived respectively from pLNs of WT and Nos2KO mice and stained with antibodies to TCR γδ (red), NOS2 (green) and counterstained with DAPI (blue). The arrow indicates a NOS2^+^ γδ T cell. Bars 10 μM. 40 X objective. The experiment was performed from WT (n = 3) and Nos2KO (n = 2) mice. (C-D) IL-2 production by γδ T cells after 4h PMA/ionomycin stimulation of pLNs from WT (n = 12) and Nos2KO (n = 12) mice. (C) Representative dot plots of IL-2 staining among γδ T cells in WT and Nos2KO mice. (D) Percentages of IL-2^+^ cells among γδ T cells are shown in left and geometric mean of fluorescence intensity (MFI) in right. Data are pooled from two experiments. Point represents individual mouse, bars are mean and box and whiskers are min to max values and median. ** *p<0*.*01*, *** *p<0*.*001* (Mann-Whitney’s test).

We have recently shown that NOS2 improves the recruitment of γδ T cells within the tumor microenvironment in a murine model of spontaneous melanoma [27]. Here we wondered whether NOS2 also impacts γδ T cell proportions at steady state. While the pools of CD4^+^ and CD8^+^ αβ T cells are similar in peripheral LNs (pLNs) of Nos2KO and WT mice (Fig 1A), the proportion and absolute number of γδ T cells were significantly reduced in pLNs of Nos2KO mice compared to WT animals (Fig 1B). Thymus and skin contained the same γδ T cell pool excluding both thymus retention and skin relocalization of γδ T cells in Nos2KO mice (Fig 1C and 1D). Moreover, similar percentages of pLN derived γδ T cells positive for annexin V were observed in both groups (Fig 1E), indicating that NOS2 deficiency did not increase γδ T cell death. To assess whether the difference of γδ T cell pool observed *in vivo* may be due to NOS2 expression by γδ T cell themselves at steady state, we performed double stainings from single cell suspensions directly *ex vivo*. As illustrated in Fig 2A, we detected few double stained cells in pLNs of WT mice, whereas no γδ T cell positive for NOS2 was found in pLNs derived from Nos2KO mice (Fig 2B). Next, we compared the ability of peripheral γδ T cells to produce IL-2 known to promote γδ T cell expansion [28]. Consistent with the reduced pool of γδ T cells in Nos2KO mice, γδ T cells from these mice produced significantly less IL-2 than their WT counterparts in response to PMA/ionomycin stimulation (Fig 2C and 2D). Both the percentage of IL2^+^ among γδ T cells and their capacity to produce IL-2 as revealed by mean fluorescence intensity (MFI) are reduced in Nos2KO mice (Fig 2D). These results suggest that NOS2 regulates the pool of γδ T cells *in vivo* through an IL-2 dependent mechanism.

### Autocrine NOS2 improves proliferation of pLN γδ T cells

**Fig 3:**
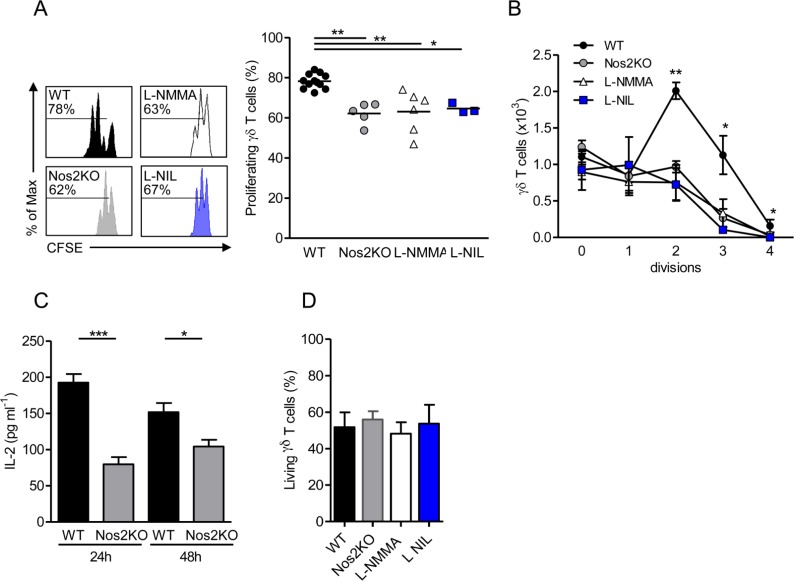
Effect of autocrine NOS2-derived NO on γδ T cell proliferation. γδ T cells sorted from pLNs of WT or Nos2KO mice and labeled with CFSE were cultured for 2 days in presence of CD3- and CD28-specific antibodies, 0,5 mM L-NIL and 10 mM L-NMMA when indicated. (A) Representative histograms of CFSE dilution. Numbers above line on CFSE plots indicate percent of proliferating cells (left). Percentages of γδ T cell proliferation undergoing division (right). (B) Number of γδ T cells by division. (C) IL-2 levels quantified in supernatants by ELISA after 24h and 48h of γδ T cell cultures. (D) Proportion of living γδ T cells after 48h culture. Data are from five independent experiments with 12 WT, 5 Nos2KO, 3 L-NIL and 6 L-NMMA replicates. Point represents individual replicate, bars mean ± SEM (except in B right mean) * *p<0*.05 ** p<0.*01*, *** *p<0*.*001* (Mann-Whitney’s test).

Next, we evaluated whether NOS2 is induced in response to T cell receptor (TCR) triggering for two days *in vitro*. We cultured cells in presence of IL-2 to expand them before the intracellular NOS2 staining and FACS analysis. γδ T cells sorted from pLNs of WT mice revealed that almost 15% of γδ T cells express NOS2 after stimulation with CD3- and CD28-specific antibodies, whereas only 2% of NOS2^+^ γδ T cells are detected in response to TCR sub-optimal stimulation with anti-CD28 (S1 Fig). Next, we analyzed the proliferation capacity of γδ T cells *in vitro*. After 48h of TCR triggering in the absence of IL-2, 78% of WT γδ T cells proliferated as determined by CFSE dilution, whereas only 62% of NOS2-deficient γδ T cells did (Fig 3A). WT γδ T cells treated with L-NMMA, a NOS inhibitor, or with L-NIL, a specific NOS2 inhibitor, proliferated to the same extent as their counterparts from Nos2KO mice (Fig 3A and 3B). These results showed that NOS2 deficiency impairs the proliferation via a direct or indirect effect. Taking into account the ability of γδ T cells to express NOS2 at steady state (Fig 2A) and that our proliferation assay is performed from purified γδ T cells, our results suggest a direct effect of endogenous NOS2 on γδ T cell proliferation (Fig 3A and 3B). In agreement with data shown in Fig 2, we quantified lower IL-2 levels in supernatants of NOS2-deficient γδ T cells after 24h and 48h of culture (Fig 3C). In addition, the decreased percentage of proliferation in NOS2-deficient cells or following L-NMMA and L-NIL treatments was not due to higher cell mortality (Fig 3D). Taken together our data suggest that NOS2 expression in γδ T cells promotes their proliferation *in vitro* that may explain the lesser γδ T cell number observed *in vivo* in Nos2 deficient mice.

### Autocrine NOS2 up-regulates glycolytic metabolism in pLN γδ T cells

**Fig 4:**
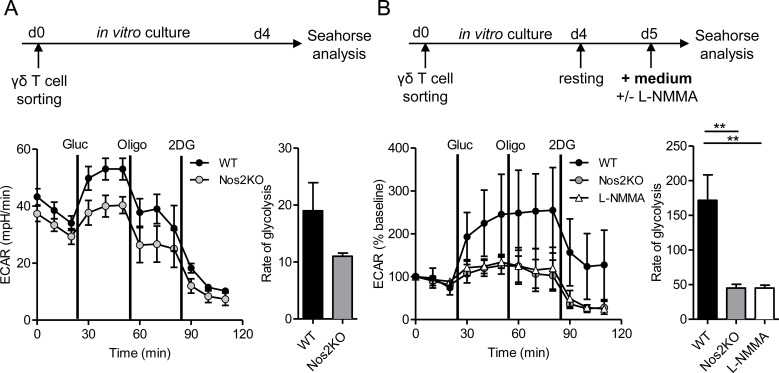
Glycolytic metabolism assessed in competent or NOS2 deficient γδ T cells. Sorted γδ T cells from pLNs of WT or Nos2KO mice were expanded *in vitro* for 4 days in presence of CD3- and CD28- specific antibodies, 15 μg/mL IL-7 and 15U/mL IL-2. Glycolytic metabolism was analyzed using a Seahorse XF-24 analyzer either directly (A), or after 18 h of resting followed by an additional 4h of stimulation with media containing 5 mM L-NMMA when indicated (B). ECAR was assessed after glucose (gluc) addition and in response to metabolic inhibitors oligomycin (oligo) and 2-Deoxy-D-glucose (2DG). Shown are time courses (A left), normalized time courses as % of baseline (B left) and calculations of rate of glycolysis (A, B right panels). Data are from one experiment with 3 (Nos2KO) and 4 (WT) replicates (A) and are pooled from three independent experiments with 7 (WT), 5 (Nos2KO) and 5 (L-NMMA) replicates (B). Mean ± SEM are shown. ** *p < 0*.*01* (Mann-Whitney’s test).

To investigate whether the reduced proliferation capacity of NOS2-deficient γδ T cells relies on a metabolic failure, we compared the metabolic phenotypes of γδ T cells from WT and Nos2KO mice. We evaluated the oxygen-consumption rate (OCR) and extracellular acidification rate (ECAR), proportional to mitochondrial respiration and aerobic glycolysis, respectively. WT and NOS2-deficient γδ T cells displayed the same OCR at basal level and after addition of metabolic inhibitors (S2 Fig), suggesting that mitochondrial respiration is NOS2 independent. Both γδ T cells displayed increased ECAR following glucose addition (Fig 4A), but the glycolysis rate was about two fold less for NOS2-deficient γδ T cells (Fig 4A). Oligomycin was added to promote maximal ECAR. However, γδ T cells, expressing NOS2 or not, did not show any ECAR increase, indicating that they had already reached their maximal glycolytic capacity after glucose addition. Because of high levels of ECAR at baseline, γδ T cells were then starved for 18h before metabolic analysis (Fig 4B). WT γδ T cells performed significantly more glycolysis than NOS2-deficient γδ T cells or L-NMMA treated WT γδ T cells (Fig 4B) revealing a role of endogenous NOS2 in the control of this metabolic pathway. Collectively, these data show that autocrine NOS2 is essential for optimal glycolysis of γδ T cells and suggest that a more efficient glycolysis in NOS2 expressing γδ T cells may contribute to their better capacity to proliferate.

### IL-2 overrides the impairment of proliferation and glycolysis in NOS2-deficient γδ T cells

**Fig 5:**
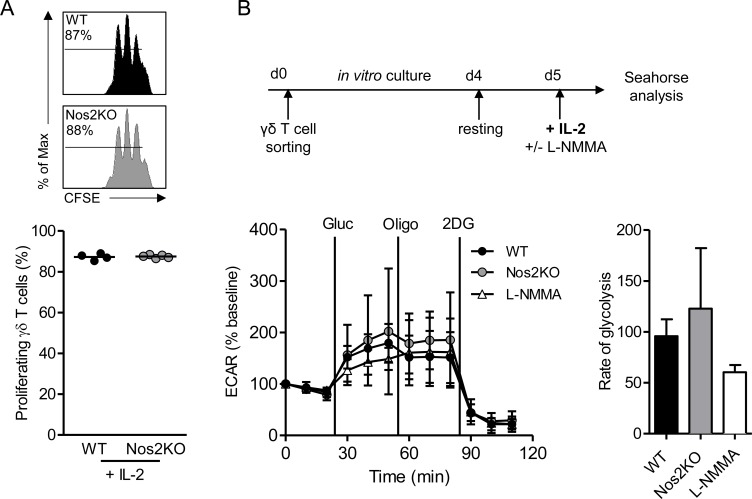
Proliferation and glycolysis of NOS2-deficient γδ T cells in presence of IL-2. (A) γδ T cells sorted from pLNs of WT and Nos2KO mice and labeled with CFSE were cultured for 2 days in presence of CD3 and CD28-specific antibodies and 15U/mL IL-2. Numbers above line on CFSE plots indicate percent of proliferating cells. Percentages of γδ T cell proliferation are shown below. Data are representative of two experiments with 4 WT and 5 Nos2KO replicates. (B) Sorted γδ T cells from pLNs were expanded *in vitro* for 4 days in presence of CD3 and CD28-specific antibodies, 15 μg/mL IL-7 and 15U/mL IL-2. ECAR was analyzed after 18h of resting followed by an additional 4h of stimulation with 15U/mL IL-2. Media contain 5 mM L-NMMA when indicated. Shown are normalized time courses as % of baseline and calculations of rate of glycolysis Data are pooled from three independent experiments with 7 (WT), 5 (Nos2KO) and 5 (L-NMMA) replicates Mean ± SEM are shown. (Mann-Whitney’s test).

To assess if the deficiency of proliferation and glycolysis in NOS2-deficient γδ T cells could be rescued, γδ T cells were respectively cultured or stimulated after resting with IL-2 (Fig 5A and 5B). NOS2-deficient γδ T cells proliferate as efficiently as WT γδ T cells after addition of exogenous IL-2 at the beginning of the culture (Fig 5A). The IL-2 stimulation globally increased ECAR at basal levels and after glucose and oligomycin addition in all conditions tested (S3 Fig). Interestingly, WT γδ T cells, L-NMMA treated WT γδ T cells, and NOS2-deficient γδ T cells displayed similar ECAR and glycolysis rates (Fig 5B), indicating that exogenous IL-2 rescued the impairment of glycolytic metabolism in NOS2-deficient γδ T cells. These results demonstrate that IL-2 reverses glycolysis and proliferation defects in NOS2-deficient γδ T cells.

## Discussion

In this study, we show that NOS2 favors IL-2 production by γδ T cells that may regulate their expansion *in vivo*. To support these results, we demonstrate the crucial role of NOS2 in promoting efficient glycolysis and proliferation of γδ T cells *in vitro*.

To our knowledge, we provide the first evidence that primary murine γδ T cells display the capacity to express NOS2 *in situ* and *in vitro* after TCR activation at steady state. Two previous studies investigated the function of autocrine NO production by human γδ T cells and produced conflicting results. The NOS3 protein, but neither NOS1 nor NOS2, was detected in γδ T cells and endogenous NOS3-derived NO was involved in γδ T cell protection from apoptosis [29]. On the other hand, NOS2 expression was found in γδ T cells upon stimulation with heat shock protein that led to their apoptosis through the mitochondrial death pathway [30]. Our study was performed on primary γδ T cells, whereas the quoted studies used γδ T cell lines. This difference may explain why we failed to observe any effect on γδ T cell apoptosis, but rather highlighted an effect on γδ T cell expansion.

Recently, we detected NOS2 expressing γδ T cells within the primary tumor of melanoma patients, but also in the primary tumor and tumor draining LNs in mice transgenic for the Ret oncogene developing a spontaneous metastatic melanoma (model Ret) [27]. γδ T cells in Ret mice lysed less efficiently tumor cells, and produced less IFN-γ than their counterparts in Ret mice deficient for NOS2. NOS2 also promoted γδ T cell polarization toward a pro-tumorigenic profile, in particular through regulating the balance between CD27^-^/CD27^+^ favoring their capacity to produce IL17. Collectively, our data indicate that NOS2 drives the expansion of γδ T cells in pLNs at steady state, but may also support, at least in part, their accumulation within the tumor microenvironment.

*In vitro* TCR activation results in the expression of NOS2 by murine CD4^+^ T cells [8]. Nevertheless and consistent with a previous study, we found a similar proportion of peripheral CD4^+^ T cells in WT and in Nos2KO mice [8]. In striking contrast, we show that NOS2 regulates the pool of peripheral γδ T cells *in vivo* by enhancing their IL-2 production. Recently, Niedbala et al found a link between NO and the up-regulation of IL-2 in Th9 cells, an αβ T cell subset, by investigating the effect of NO donor on Th9 polarization *in vitro*. Indeed, nitrosylation of cysteine residues induces a signaling cascade leading to higher IL-2 production by Th9 cells [31]. Although no evidence for Th9 cell-intrinsic expression and function of NOS2 was provided, it is tempting from these data and ours to envisage that such a NOS2 dependent post-translational modification drives IL-2 production in γδ T cells.

Proliferating T cells require adaptation of their metabolic programs to provide sufficient biosynthetic precursors and adequate energy. In particular, activated αβ T lymphocytes up-regulate aerobic glycolysis to rapidly grow and divide [32]. Here, we found that endogenous NOS2 enhanced glycolysis in γδ T cells consistent with their efficient ability to proliferate *in vitro*. The effects of NOS2 on immune cell metabolism remain poorly described. Nevertheless, NOS2-derived NO was identified as a metabolic regulator in inflammatory DC [33]. Autocrine NO production by DC leads to a switch towards glycolysis as a consequence of inhibition of mitochondrial respiration. Glycolysis is linked to availability of nutrients such as glucose and depends on glucose uptake. We hypothesized that NOS2 defective γδ T cells express fewer glucose transporters than WT γδ T cells, resulting in less capacity for glycolysis and subsequently reduced proliferation. To support this hypothesis, NO has been shown to enhance glucose uptake in the HEK293T cell line via up-regulation of the glucose transporter (GLUT) 3 [34]. Moreover, we show that the addition of exogenous IL-2 restored the rate of glycolysis and proliferation in NOS2 defective γδ T cells. Consistent with our results, IL-2 promotes GLUT1 expression and glucose uptake in activated αβ T cells [35–37]. Thus, NOS2, by up-regulating IL-2, could maximize expression of GLUT on γδ T cells resulting in their efficient proliferation *in vivo*. These hypotheses need to be investigated elsewhere.

This study show for the first time that NOS2 affects metabolism of γδ T cells. Metabolic regulations of immune cells are closely linked to their functions and changes in metabolism have been shown to enhance or suppress T cell functions and fate [36]. Understanding the metabolic regulations of αβ and γδ T cells appears to be a critical step toward the development of more efficient immunotherapy strategies based on these cells [38].

## Supporting Information

S1 Fig Evaluation of NOS2 expression by γδ T cells after TCR triggering *in vitro*. Sorted γδ T cells from pLNs of WT mice were cultured for 2 days in presence of 30 U/mL IL-2 and CD3- and CD28-specific antibodies when indicated (n = 4 replicates each condition). Cells were stained for NOS2 and a viability marker. Flow cytometry representative of NOS2 staining (left) and percentages of NOS2^+^ γδ T cells among living cells (right) are shown. Numbers above line indicate percent of NOS2^+^ γδ T cells. * *p<0*.*05* (Mann-Whitney’s test).
(TIF)

S2 Fig Evaluation of mitochondrial respiration in γδ T cells competent or deficient for NOS2. Sorted γδ T cells from pLNs of WT and Nos2KO mice were expanded *in vitro* for 4 days in presence of CD3 and CD28- specific antibodies, 15 μg/mL IL-7 and 15U/mL IL-2. Metabolism was analyzed using a Seahorse XF-24 analyzer. OCR was assessed in response to mitochondrial inhibitors: oligomycin (oligo), Carbonyl cyanide 4—(trifluoromethoxy) phenylhydrazone (FCCP), and rotenone and antimycin A (Rot/AntiA). Shown are time courses. Data are from one experiment with 3 (Nos2KO) and 4 (WT) replicates.
(TIF)

S3 Fig Comparison of γδ T cell glycolysis before and after addition of exogenous source of IL-2. γδ T cells sorted from pLNs of WT and Nos2KO mice were expanded *in vitro* for 4 days in presence of CD3 and CD28-specific antibodies, 15 μg/mL IL-7 and 15U/mL IL-2. Glycolytic metabolism analysis was performed after 18 h of resting following by an additional 4 h of stimulation with media containing 5mM L-NMMA and/or 15U/mL IL-2 when indicated. ECAR was assessed after adding glucose and in response to metabolic inhibitors oligo and 2DG. Time courses are pooled from three independent experiments.
(TIF)
