# Supplementary figures and images for: Nitric Oxide Synthase 2 Improves Proliferation and Glycolysis of Peripheral γδ T Cells
Source: PLoS One. 2016 Nov 3;11(11):e0165639. doi: 10.1371/journal.pone.0165639 (PMC5094591; doi:10.1371/journal.pone.0165639)

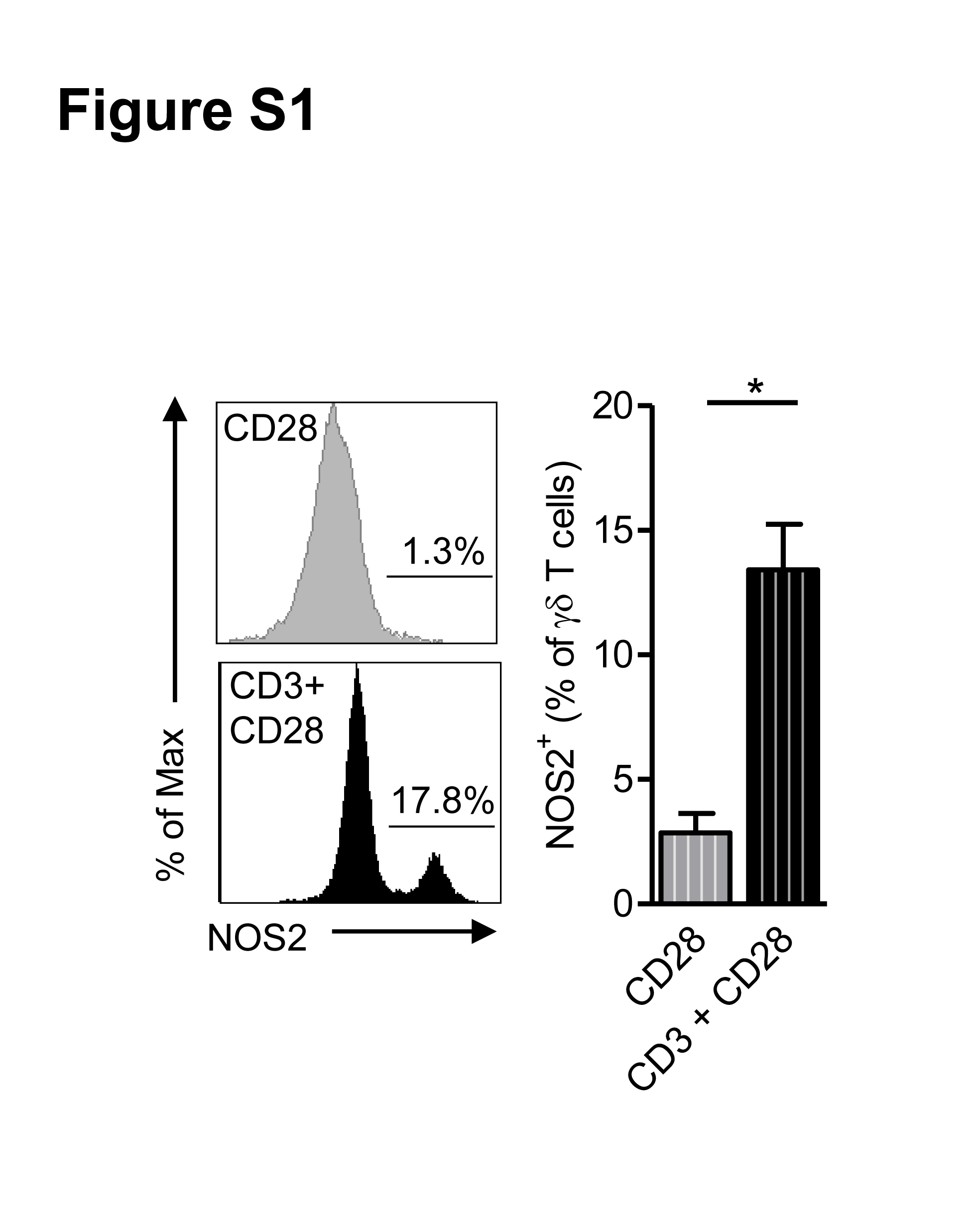

Supplement: S1 Fig — Sorted γδ T cells from pLNs of WT mice were cultured for 2 days in presence of 30 U/mL IL-2 and CD3- and CD28-specific antibodies when indicated (n = 4 replicates each condition). Cells were stained for NOS2 and a viability marker. Flow cytometry representative of NOS2 staining (left) and percentages of NOS2+ γδ T cells among living cells (right) are shown. Numbers above line indicate percent of NOS2+ γδ T cells. * p<0.05 (Mann-Whitney’s test). (TIF) [file pone.0165639.s001.tif]

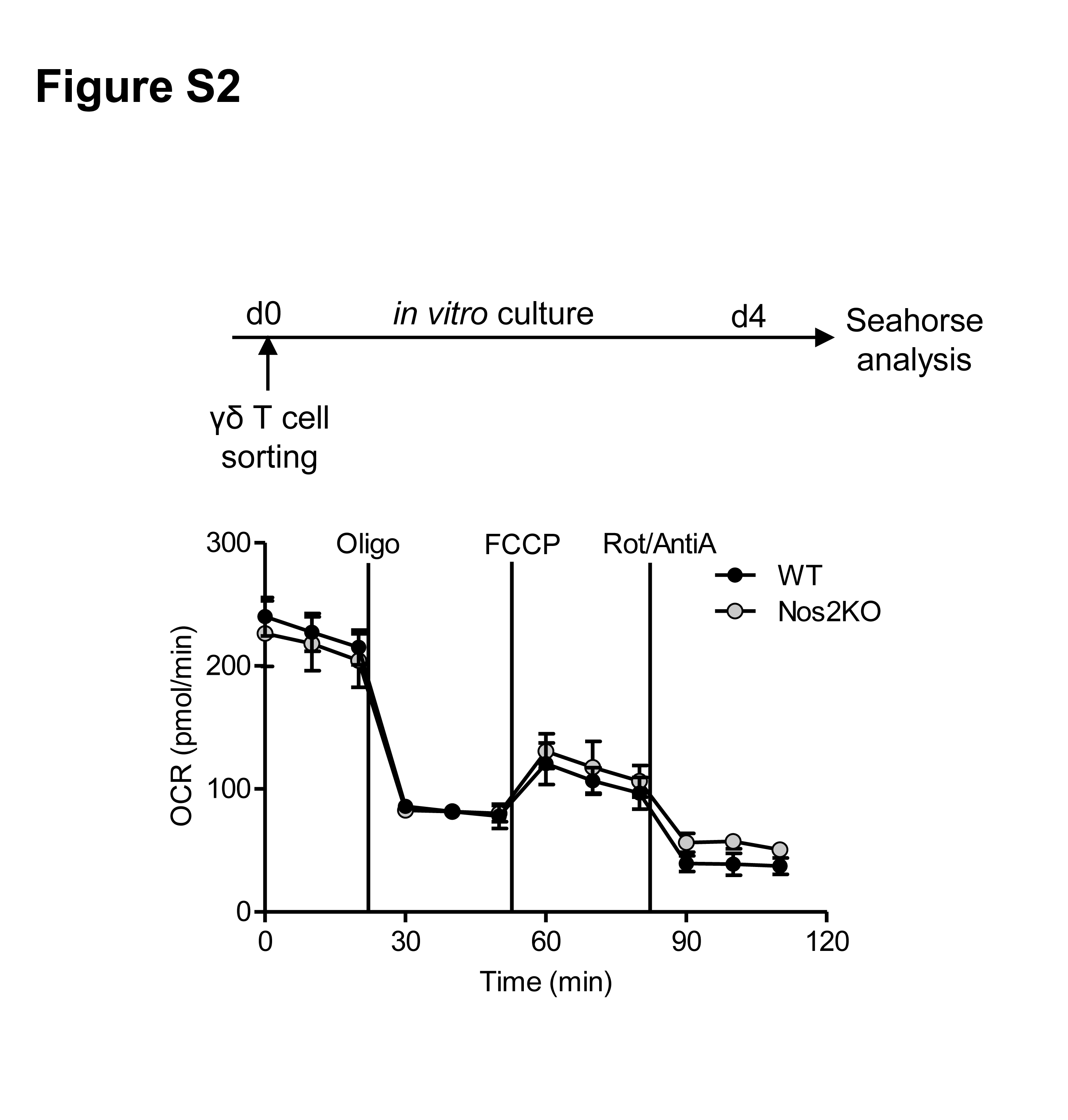

Supplement: S2 Fig — Sorted γδ T cells from pLNs of WT and Nos2KO mice were expanded in vitro for 4 days in presence of CD3 and CD28- specific antibodies, 15 μg/mL IL-7 and 15U/mL IL-2. Metabolism was analyzed using a Seahorse XF-24 analyzer. OCR was assessed in response to mitochondrial inhibitors: oligomycin (oligo), Carbonyl cyanide 4—(trifluoromethoxy) phenylhydrazone (FCCP), and rotenone and antimycin A (Rot/AntiA). Shown are time courses. Data are from one experiment with 3 (Nos2KO) and 4 (WT) replicates. (TIF) [file pone.0165639.s002.tif]

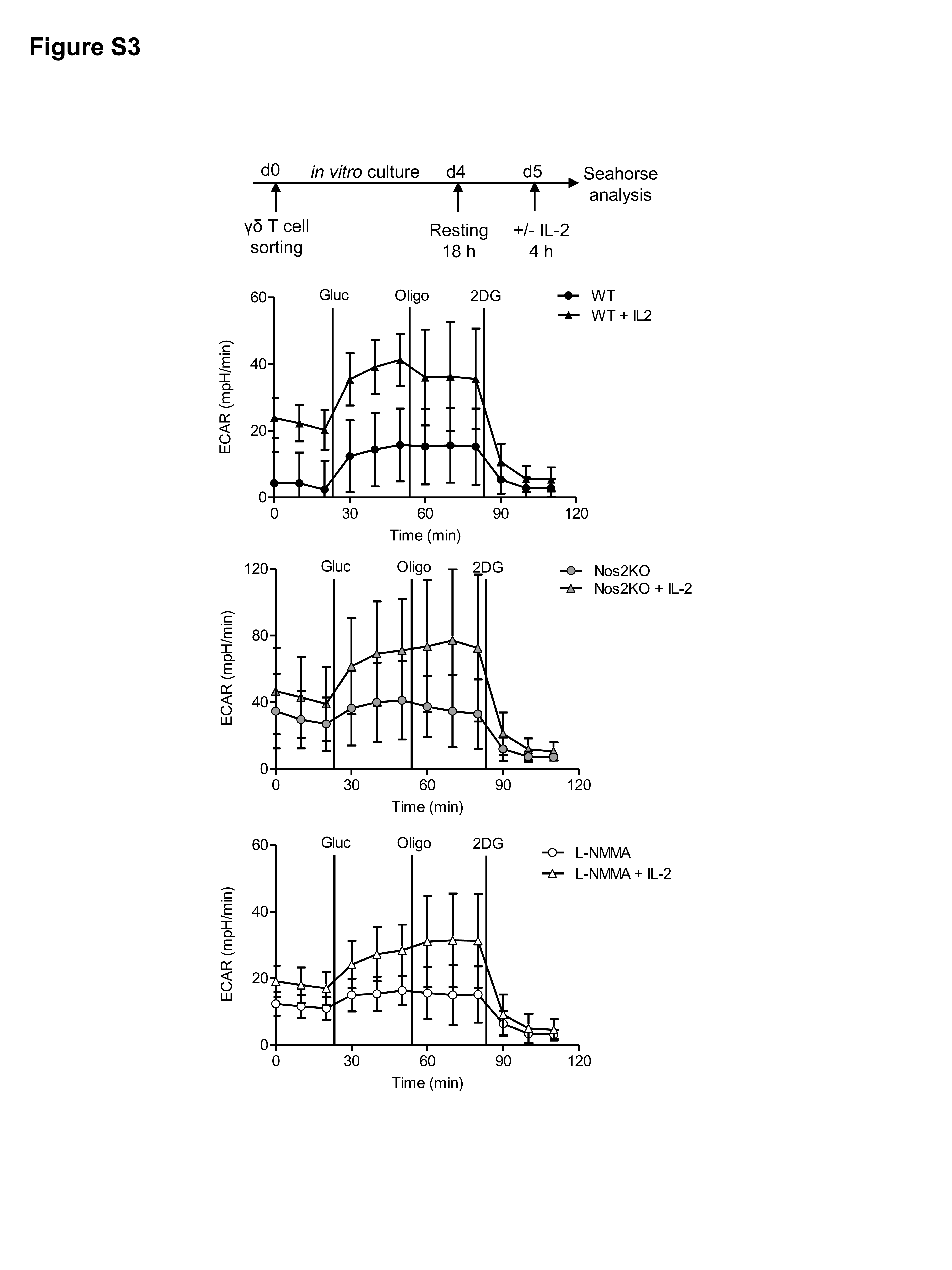

Supplement: S3 Fig — γδ T cells sorted from pLNs of WT and Nos2KO mice were expanded in vitro for 4 days in presence of CD3 and CD28-specific antibodies, 15 μg/mL IL-7 and 15U/mL IL-2. Glycolytic metabolism analysis was performed after 18 h of resting following by an additional 4 h of stimulation with media containing 5mM L-NMMA and/or 15U/mL IL-2 when indicated. ECAR was assessed after adding glucose and in response to metabolic inhibitors oligo and 2DG. Time courses are pooled from three independent experiments. (TIF) [file pone.0165639.s003.tif]
